# Host Defense Peptides from Asian Frogs as Potential Clinical Therapies

**DOI:** 10.3390/antibiotics4020136

**Published:** 2015-03-30

**Authors:** Vineeth T.V. Kumar, David Holthausen, Joshy Jacob, Sanil George

**Affiliations:** 1 Molecular Ecology Lab, Rajiv Gandhi Centre for Biotechnology (RGCB), Thiruvananthapuram, Kerala 695014, India; E-Mail: vineethkumartv@rgcb.res.in; 2 Emory Vaccine Center, Department of Microbiology and Immunology, Emory University, Yerkes National Primate Research Center, 954 Gatewood Rd, Atlanta, GA 30329, USA; E-Mail: dholtha@emory.edu

**Keywords:** Host defense peptides, Asian frog, Multi-drug resistance, Anti-microbial peptides

## Abstract

Host defense peptides (HDPs) are currently major focal points of medical research as infectious microbes are gaining resistance to existing drugs. They are effective against multi-drug resistant pathogens due to their unique primary target, biological membranes, and their peculiar mode of action. Even though HDPs from 60 Asian frog species belonging to 15 genera have been characterized, research into these peptides is at a very early stage. The purpose of this review is to showcase the status of peptide research in Asia. Here we provide a summary of HDPs from Asian frogs.

## 1. Introduction

The origins of the study into host defense peptides began with the pioneering studies of Vittorio Erspamer, who discovered biogenic amines and peptides secreted by the amphibian skin. The field later diversified when Zasloff isolated magainin from *Xenopus laevis* [1,2]. In our current era of multi-drug resistant bacterial strains, discovering new and effective treatments to replace traditional antibiotics is a critical area of research. Host defense peptides (HDPs), such as those being found in the skin of Asian frogs, have shown effectiveness in killing both gram-negative and gram-positive bacteria, as well as some viruses and cancers, without any of the drawbacks of antibiotic resistance [3]. These properties make them viable candidates as the next wave of therapies against infectious and non-infectious diseases that are the leading cause of death in developing countries.

Research on how these anti-microbial peptides function is still in the early stages. Most of the HDPs are typically cationic, a feature which allows them to interact with the negatively charged bacterial and cancer cell membranes. This cationic nature allows some peptides to actually penetrate the surface of pathogens as a means of killing them [4]. Asia is well known for its diverse amphibian populations and has been a resource for novel HDPs. Recently, potent HDPs have been isolated from amphibians located in temperate regions (traditionally believed to have low biodiversity) and have proven to be efficient in combating many clinical pathogens, though research into these peptides is still at a very early stage. We hope as we continue to discover new peptides in this region we will also find more potential candidates for novel therapeutics.

## 2. Diversity of HDPs Found in the Skin Secretion of Asian Frogs

**Figure A1:**
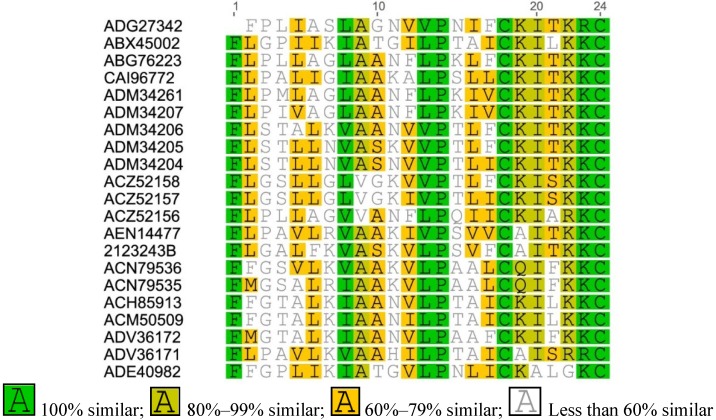
Multiple sequence alignment of Brevinin-1 from Asian frogs.

**Figure A2:**
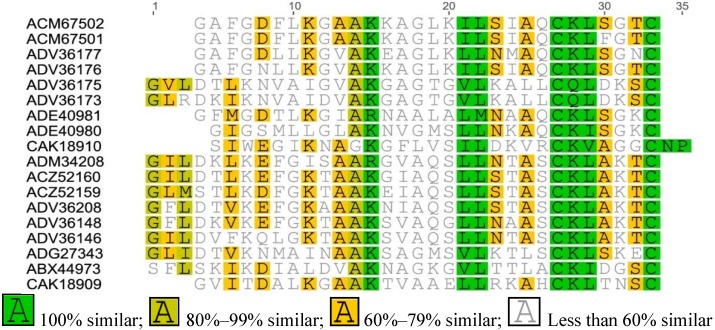
Multiple sequence alignment of Brevinin-2 from Asian frogs.

**Figure A3:**
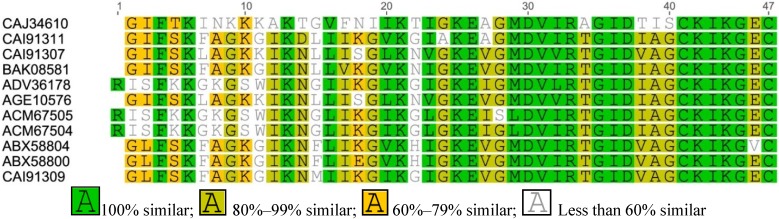
Multiple sequence alignment of Esculetin-1 from Asian frogs.

**Figure A4:**
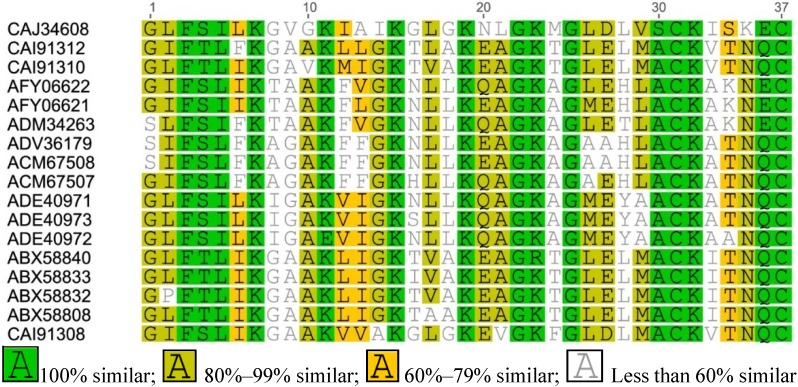
Multiple sequence alignment of Esculetin-2 from Asian frogs.

In the tropics, especially in the rain forests of Asia, effective host defense peptides have helped the frogs to survive within their environment and attain a rich diversity. However with the alarming rate of decline in amphibians globally, many identified and unidentified species have become extinct before researchers ever had a chance to isolate their HDPs for study [5]. Those peptides that have been isolated show a great degree of variability—even within a single genus, different species produce varied repertoires. These differences can be correlated to habitat specific challenges endured by each species, and how these frogs uniquely evolved alongside their environment [6]. Even within the same frog, expression of several members of a particular family of HDP is observed. The variation in these peptide sequences is suggestive evidence for ancestral gene duplication [7]. No two peptides with identical amino acid sequences have been reported even from closely related species. Such diversity provides broad-spectrum protection against the large pool of microbes/predators in their habitat (Figure A1, Figure A2, Figure A3 and Figure A4) [8]. Transcriptomic and peptidomic analysis revealed that genera will have HDP families in common, apart from this, some unique peptides, such as neuroendocrine and smooth muscle active, are reported that serve a particular purpose in that habitat e.g., bradykinin related peptides (BRPs) and Trypsin inhibitors from Odorrana genus. Here we review the HDP families reported from Asian frogs. Despite the wealth of biodiversity, attempts to characterize HDPs from Asian frogs have been limited. In the present medical scenario there is an urgent need to uncover the hidden peptides that may lead to developing new and potentially crucial therapeutic agents.

### 2.1. Amolops

Genus Amolops, which belongs to the family Ranidae, is endemic to China. Eight species of frogs from this genus have been studied and their HDPs isolated (Table 1 and Table 2). The most common peptides isolated are of the Brevenin 1 and 2, Esculentin 2, Palustrin 2, and Temporin families, and all of them have been shown to be active against gram-positive and -negative bacteria, fungi, and cancer cells [9,10,11,12,13,14]. Cytotoxicity analysis against K562 and HT29 of Palustrin 2 from *A. jindongensis* skin revealed IC_50_ values between 57–58 µM [11].

**Table 1:** List of peptides identified from the skin secretion of Asian frogs.

		Peptides isolated from the respective frog species: numbers indicate paralogs of each peptide family						
**Genus: Amolops**		**Brevinin-1**	**Brevinin-2**	**Esculentin-2**	**Palustrin-2**	**Temporin**	**Novel family of Peptides**	**Ref**
1	*A. chunganensis*	5	1	1	1	5		[9,10,11,12,13,14,15,16,17]
2	*A. hainanensis*		2		1		Amylopin-1: 3 Amylopin-6: 1 Hainanenin 1-5,5 families: 5	
3	*A. jindongenensis*	1		2	2		Jindongnenin: 1	
4	*A. loloensis*	4		2		11	Amylopin 1-2,2 families: 2 Amylopkinin: 1	
5	*A. lifanensis*	3		1				
6	*A. ricketti*	3	2					
7	*A. torrentis*		1					
8	*A. wuyiensis*						Amylopkinin: 2	
**Genus: Glandirana**		**Brevinin-1**	**Brevinin-2**	**Esculentin-2**				**Ref**
1	*G. rugosa*	2 (Gaegurin 5–6)	6 (Gaegurin 1–3 Rugosin A,B,C)					[13,18,19,20]
2	*G. emeljanovi*	2 (Gaegurin 5–6)		1 (Gaegurin 5–6)				
**Genus: Hylarana**		**Brevinin-1**	**Brevinin-2**	**Esculentin-1**	**Esculentin-2**	**Temporin**	**Novel family of Peptides**	**Ref**
1	*H. erythrea*	3	2		4	1		[8,21,22,23,24,25,26,27,28,29,30,31,32,33,34,35,36]
2	*H. guentheri*	2	6			6	Guentherin: 1 Bradykinin BRP: 12 *	
3	*H. latuouchii*	4	3	2	2	6	Palustrin-2	
4	*H. luctiosa*		4	1	1	2	Palustrin-2: 2	
5	*H. nigrovittata*	7 (7 **Gaegurins**)	9 (9 **Rugosins**)			3	Nigroain: 15 Ranakinin N: 1 Cholycytokinin: 1	
6	*H. picturata*	2	5			1		
7	*H. signata*	5	4			2	Palustrin-2	
8	*H. spinulosa*	2	4	1	2	5	Spinulosain: 1 Ranatuerin: 1 Nigroain: 5 Odorranain: 1 Ranacyclin: 1	
9	*H. temporalis*	1	2		3		Hylaranakinin: 2	
**Genus: Odorrana**		**Brevinin-1**	**Brevinin-2**	**Esculentin-1**	**Esculentin-2**	**Nigrocin-2**	**Novel family of Peptides**	**Ref**
1	*O. grahamii*	2	4	2	4	4	Takykinin: 2 TrypsinInhibitor: 1, BRP: 3 * BLP: 5 * Odorrnalectin: 1 Palustrin-2: 1 Grahamin 1–2: 2 families: 2 Odorranain: 27	[14,28,31,37,38,39,40,41,42,43,44,45,46,47,48,49,50,51,52,53,54,55,56,57,58]

2	*O. hananensis*	2	2				Odorrnain: 2 Temporin: 2	
3	*O. hejiangensis*						TrypsinInhibitor: 1	
4	*O. hossi*	2	2	1	1	2		
5	*O. ishikawae*	1	3	2	1	5	Ishikawain 1–8, 8 families: 8 Palustrin 2: 3 Odorranain: 2	
6	*O. jingdongensis*	3	1		2	2		
7	*O. livida*						Lividin 1–4, 4 families: 4	
8	*O. schmakeri*	3	3	1	1		BRP: 7 *	
9	*O. tiannanensis*	3		1	2	1	Mararetaein: 2 Pleurain: 1 Tiannenensin: 1 Odorranain: 10	
10	*O. versablis*	2		2	2		Ranatuerins: 2 Temporin: 1 TrypsinInhibitor: 1	
**Genus: Pelophylax**		**Brevinin-1**	**Brevinin-2**	**Esculentin-1**	**Esculentin-2**	**Novel family of Peptides**		**Ref**
1	*P. plancyi*		3					[13,20,56,59,60]
2	*P. porosus*		1					
3	*P. chosenicus*		1					
4	*P. fukienensis*	1		1	1	Pelophylaxin 1–4, 4 families Ranakinestatin: 1		
5	*P. nigromaculata*		1 (Nigrocin-1)	2		Nigocin-2: 1		
**Genus: Rana**		**Brevinin-1**	**Brevinin-2**	**Temporin-1**	**Ranatuerin-2**	**Novel family of Peptides**		**Ref**
1	*R. amurensis*	3 (Amurin 1–3:3 families)			2			[20,61,62,63,64,65,66,67,68,69,70,71,72,73,74,75,76,77,78,79,80]
2	*R brevipoda porsa*	1	1					
3	*R. chaochioensis*					Japonicin-2: 4		
4	*R. chensinensis*	6	3	11		RCSK 1–4, 4 families: 4 * Chensinin 1–4, 4 families: 7 Japonicin-1: 1 D-1CDYa: 1 **		
5	*R. dybowskii*	18 (9 Dybowskins)	7	3		Japonicin-1: 1		
6	*R. japonica*			1		Japonicin-1: 1 Japonicin-2: 1		
7	*R. okinavana*	4			1			
8	*R. ornativentris*		2	7	4	Palustrin: 1		
9	*R. pirica*	1	5	2	1			
10	*R. pleuradan*					Pleurain: 2		
11	*R. sakurai*		2	4	1	MRP: 1 *, BRP: 1 *		
12	*R. shuchinae*					Shuchin 1–5, 5 families: 5		
13	*R. tagoi*	1		1		MRP: 1 *		
14	*R. tagoiokiensis*	2		2	2			
15	*R. tsushimensis*	1	1	4				
**Genus: Clinotarsus**								**Ref**
1	*C. curtipes*	Brevinin-1: 5						[81]
						
**Genus: Fejervaria**								
1	*F. carcrivora*	Tigerinin: 2						[82]
						
**Genus: Hoplobatracus**								
1	*H. rugulosus*	Tigerinin-1: 1						[83,84,85]
2	*H. tigerinus*	Tigerinin 1–4, 4 families: 4						
**Genus: Hyla**								**Ref**
1	*H. annectans*	Annotoxin: 1						[86]
**Genus: Limnonectes**								
1	*L. fujianensis*	Limnonectins: 2						[87]
**Genus: Nanorana**								
1	*N. parkeri*	Japonicin: 2 Parkerin: 1						[88]
**Genus: Rhacophorus**								
1	*R. duboisi*	Polypedarelaxin: 1 Polypedatein: 1						[89,90]
2	*R. scheglii*	Histone 2B						
**Genus: Sanguirana**								
1	*S. varians*	BLP: 1 *						[91]
**Genus: Euphlyctis**								
1	*E. hexadactylus*	Crude skin extract: peptidesnot characterized						[92]

* Abbreviations used in the table: BRP—Bradykinin like peptide, BLP—Bombesisn like peptide, RCSK—*R. chensinensis* skin kinogen, MRP—Mellitin related peptide. Paralogs: Peptides of a particular family with little sequence homology found within a species, Orthologs: Particular family of peptides found withi two different species or genuses. ** CDY in the peptide D-1CDYa was used to indicate that the peptides were obtained from Chinese frog (C) found in Dongbei Region (D, northeast), city of Xifeng (Y).

**Table 2:** List of peptides identified from the skin secretion of Asian frogs with their corresponding MIC and IC_50_/LD_50_ values.

		MIC and IC_50_/LD_50_ values					
**Genus: Amolops**		**Brevinin-1**	**Brevinin-2**	**Esculentin-2**	**Palustrin-2**	**Temporin**	**Novel family of Peptides**
1	*A. chunganensis*	G+: 0.5–75 µM G−: 4–150 µM F: 4–150 µM LD50: 15–150 µM	G+: 2–75 µM G−: 2–150 µM F: 9 µM LD50: 75 µM	G+: 1–19 µM G−: 75–150 µM F: 4.5 µM LD50: 75 µM	G+: 2–150 µM G−: 4–10 µM F: 4.5 µM LD50: 75 µM	G+: 4–150 µM G−: 75 µM F: 9–150 µM LD50: 150–200 µM	
2	*A. hainanensis*		Unknown		Unknown		Amylopins: G+: 37–75 µg/mL G−: no active Hainanenin: G+: 4–40 µM G−: 4–75 µM F: 2–75 µM
3	*A. jindongenensis*	Unknown		Unknown	G+: 20 µM G−: 13–50 µM F: not active IC50: 57–58 µM (K562 & HT29 cell lines)		Jindongnenin:G+: 17–60 µM G−: 10–40 µM F: 60 µM
4	*A. loloensis*	G+: 5 µg/mL G−: 2–7 µg/mL F: not active IC50: 58 µg/mL (HepG2 cell line)		G+: 1–8 µg/mL G−: 7–50 µg/mL F: 2–22 µg/mL		G+: 1–75 µg/mL G−: 1–75 µg/mL F: 1–25 µg/mL IC50: 77 µg/mL (HepG2 cell line)	Amylopin: G+: 37–75 µg/mL G−: no active Amylopkinin: Smooth muscle active peptide
5	*A. lifanensis*	Unknown		Unknown			
6	*A. ricketti*	G+: 3–25 µg/mL G−: 12.5 µg/mL F: 100–200 µg/mL LD50: 100–200 µg/mL	G+: 1–200 µg/mL G−: 6–15 µg/mL F: 200 µg/mL				
7	*A. torrentis*		Unknown				
8	*A. wuyiensis*						Amylopkinin: Smooth muscle active peptide
**Genus: Glandirana**		**Brevinin-1**	**Brevinin-2**	**Esculentin-2**			
1	*G. rugosa*	Unknown	G+: 6–50 µg/mL G−: 12.5–100 µg/mL				
2	*G. emeljanovi*	Unknown		Unknown			
**Genus: Hylarana**		**Brevinin-1**	**Brevinin-2**	**Esculentin-1**	**Esculentin-2**	**Temporin**	**Novel family of Peptides**
1	*H. erythrea*	Unknown	G+: 12.5 µM G−: 12.5 µM F: 50 µM LD50: 280 µM		Unknown	Unknown	
2	*H. guentheri*	Unknown	G+: 3–6 µM G−: 2–6 µM F: not active LD50: 280 µM			G+: 30–50 µM G−: not active F: not active	Guentherin G+: 33.5 µg/mL Bradykinin, BRP: smooth muscle active peptide
3	*H. latuouchii*	G+: 6–10 µg/mL G−: 12.5 µg/mL F: 100–200 µg/mL LD50: 100–200 µg/mL	G+: 0.5–8 µg/mL G−: 0.5–130 µg/mL F: not active LD50: 400–600 µg/mL	G+: 0.6–10 µg/mL G−: 0.6–10 µg/mL F: 80 µg/mL LD50: 500 µg/mL	G+: 30–60 µg/mL G−: 6–15 µg/mL F: not active LD50: 500 µg/mL	G+: 6–10 µg/mL G−: not active F: not active LD50: 40 µg/mL	Palustrin: G+: 1–14 µg/mL G−: not active F: not active LD50: 220 µg/mL
4	*H. luctiosa*		Unknown	Unknown	G+: 4 µM G−: 32 µM	G+: 32 µM G−: 128 µM	Palustrin: G+: 1–14 µg/mL G−: not active F: not active LD50: 220 µg/mL
5	*H. nigrovittata*	G+: 1–65 µg/mL G−: 18–40 µg/mL F: 2–5 µg/mL	G+: 4–20 µg/mL G−: 25–100 µg/mL F: 5–20 µg/mL			G+: 3–9 µg/mL G−: 4–15 µg/mL F: 3–9 µg/mL	Nigroain:G+: 9–50 µg/mL G−: 25–110 µg/mL F: 2–4 µg/mL Ranakinin N, Cholycytokinin: smooth muscle active peptides
6	*H. picturata*	G+: 3 µM G−: 24 µM	G+: 9–18 µM G−: 9–72 µM			Unknown	
7	*H. signata*	Unknown	Unknown			Unknown	Palustrin G+: 1–14 µM
8	*H. spinulosa*	G+: 3–100 µM G−: 100–400 µM F: 12.5 µM	G+: 3–200 µM G−: 3–400 µM F: 100–400 µM	Unknown	G+: 6–200 µM G−: 12–400 µM F: not active	G+: 6–25 µM G−: not active F: 100–400 µM	Spinulosain,Ranatuerin,Nigroain,Odorranain, Ranacyclin:Unknown
9	*H. temporalis*	G+: 100–150 µg/mL G−: 30–150 µg/mL	G+: 40–150 µg/mL G−: 20–150 µg/mL		Unknown		Hylaranakinin:Unknown
**Genus: Odorrana**		**Brevinin-1**	**Brevinin-2**	**Esculentin-1**	**Esculentin-2**	**Nigrocin-2**	**Novel family of Peptides**
1	*O. grahamii*	Unknown	Unknown	Unknown	Unknown	G+: 9–100 µg/mL G−: 4–100 µg/mL F: 1–10 µg/mL	Takykinin,TrypsinInhibitor, BRP,BLP: smooth muscle active peptides Odorrnalectin: drug targeting Grahamin: G+: 2.5 µg/mL G−: 1–8 µg/mL F: 7.5 µg/mL Palustrin: G+: 12–100 µM G−: 100 µM F: 100 µM Odorranain: G+: 2–90 µg/mL G−: 3–50 µg/mL F: 1–50 µg/mL
2	*O. hananensis*	G+: 1–150 µM G−: 9–150 µM F: 1–10 µM LD50: 75 µM	G+: 9–150 µM G−: 9–10 µM F: 19–40 µM LD50: 300 µM				Odorranain: Unknown Temporin: G+: 2–150 µM G−: 30–75 µM F: 9–75 µM LD50: 75–300 µM
3	*O. hejiangensis*						Trypsin Inhibitor: smooth muscle active peptide
4	*O. hossi*	G+: 3 µM G−: 24–50 µM	G+: 18 µM G−: 36 µM	G+: 12 µM G−: 12 µM	G+: 16 µM G−: 32 µM	G+: 25–60 µM G−: 10–30 µM	

5	*O. ishikawae*	G+: 6–100 µM G−: not active F: 50 µM	G+: 6–100 µM G−: 12–50 µM F: not active	G+: 3–25 µM G−: 3–12 µM F: 50 µM	G+: 3–25 µM G−: 12.5 µM F: 100 µM	G+: 3–15 µM G−: 25–50 µM F: 50 µM	Ishikawain: Unknown Palustrin-2: G+: 12–100 µM G−: 100 µM F: 100 µM Odorranain: Unknown
6	*O. jingdongensis*	G+: 6–15 µM G−: 25–50 µM F: 50µM	G+: 19 µM G−: 38 µM F: 19 µM		G+: 8 µM G−: 34 µM F: not active	G+: 8–16 µM G−: 15–16 µM F: 30–70 µM	
7	*O. livida*						Lividin 1–4: Unknown
8	*O. schmakeri*	Unknown	Unknown	Unknown	Unknown		BRP: 7
9	*O. tiannanensis*	Unknown		Unknown	Unknown	Unknown	Mararetaein, Pleurain, Odorranain: Unknown Tiannenensin: G+: 75 µM F:>100 µM
10	*O. versablis*	Unknown		Unknown	Unknown		Ranatuerins, Temporin, Trypsininhibitor: Unknown
**Genus: Pelophylax**		**Brevinin-1**	**Brevinin-2**	**Esculentin-1**	**Esculentin-2**		**Novel family of Peptides**
1	*P. plancyi*		Unknown				
2	*P. porosus*		Unknown				
3	*P. chosenicus*		Unknown				
4	*P. fukienensis*	Unknown		Unknown	Unknown		Pelophylaxin: Unknown Ranakinestatin: bradykinin antagonist
5	*P. nigromaculata*		Unknown				Nigocin-2 G+: 2.5 µg/mL G−: 10–100 µg/mL F: 150 µg/mL
**Genus: Rana**		**Brevinin-1**	**Brevinin-2**	**Temporin-1**	**Ranatuerin-2**		**Novel family of Peptides**
1	*R. amurensis*	Unknown			2		
2	*R. brevipoda porsa*	G+: 8 µg/mL G−: 34 µg/mL	G+: 8 µg/mL G−: 4 µg/mL				
3	*R. chaochioensis*						JaponicinG+: 25–100 µg/mL G−: 12–100 µg/mL
4	*R. chensinensis*	G+: 12.5 µM G−: 25 µM HC50: 180–200 µM	3	G+: 100 µM G−: 100 µM IC50: 30–60 µM (Mcf 7 breast cancer cell line) LD50: 100 µM			RCSK 1–4, Chensinin 1–4, Japonicin-1, D-1CDYa: G+: 6–8 µM G−: 3–5 µM HC50: 450 µM
5	*R. dybowskii*	G+: 12.5 µM G−: 25 µM HC50: 125 µM	G+: 15–30 µM G−: 15–30 µM	G+: 60–100 µM G−: 60–100 µM HC50: 180 µM			Japonicin-1:G+: 100 µM G−: 25 µM HC50: 300 µM
6	*R. japonica*			G+: >100 µM G−: >100 µM			Japonicin-1:G+: >100 µM G−: 30 µM Japonicin-2:
							G+: 20 µM G−: 12 µM
7	*R. okinavana*	G+: 12.5 µM G−: 6–12.5 µM F: not active			G+: 50 µM G−: 12.5 µM F: 100 µM		
8	*R. ornativentris*		Unknown	G+: 200 µM F: 200 µM	Unknown		Palustrin: Unknown
9	*R. pirica*	G+: 13µM G−: not active F: 100 µM HC50: 7 µM	G+: 25 µM G−: 3–12 µM F: 100 µM HC50: 50 µM	G+: 100 µM G−: not active F: 100 µM HC50: 300 µM	G+: 100 µM G−: not active F: 100 µM HC50: 150 µM		
10	*R. pleuradan*						Pleurain: G+: 15–30 µg/mL G−: 60–120 µg/mL F: 30 µg/mL
11	*R. sakurai*		G+: >50 µM G−: 3 µM F: not active	G+: 25 µM G−:>50 µM F: >50 µM	G+: >50 µM G−: 50 µM F: >50 µM		MRP (AR 23), BRP: Smooth muscle active peptides
12	*R. shuchinae*						Shuchin G+: 6–15 µg/mL G−: 3–50 µg/mL F: 6.25 µg/mL
13	*R. tagoi*	Unknown		G+: 10–40 µM			MRP (AR23): G+: 2–20 µM
14	*R. tagoiokiensis*	G+: 5 µM G−: 20 µM F: 20 µM		G+: 10 µM G−: 160 µM F: 80µM	G+: 160 µM G−: 80 µM F: 160 µM		
15	*R. tsushimensis*	G+: 12–25 µM G−: 25–100 µM F: 50 µM LD50: 12 µM	G+: 5 µM G−: 20 µM F: 20 µM LD50: 100 µM	Unknown			
**Genus: Clinotarsus**							
1	*C. curtipes*	Brevinin-1 G+: 6–100 µg/mL G−: 7–60 µg/mL					
**Genus*:* Fejervaria**							
1	*F. carcrivora*	Tigerinin 2G+: 20–80 µg/mL G−: 10–40 µg/mL F: 80–180 µg/mL					
**Genus*:* Hoplobatracus**							
1	*H. rugulosus*	Tigerinin 1: Insulin releasing peptide					
2	*H. tigerinus*	Tigerinin 1: G+: 20–50 µg/mL G−: 20–100 µg/mL					
**Genus *:* Hyla**							
1	*H. annectans*	Annotoxin-1: Inhibitor of tetradotoxin sensitive sodium channel					
**Genus: Limnonectes**							
1	*L. fujianensis*	Limnonectin-2: G+: not active G−: 35–70 µM LD50: 160 µM					
**Genus: Nanorana**							
1	*N. parkeri*	Japonicin:G+: 9–40 µg/mL G−: >100 µg/mL Parkerin: G+: 37.5 µg/mL G−: 37–100 µg/mL					
**Genus: Rhacophorus**							
1	*R. duboisi*	Polypedarelaxin: Smooth muscle active peptide Polypedatein: Unknown					
2	*R. Scheglii*	Histone 2B: Unknown					
**Genus: Sanguirana**							
1	*S. varians*	BLP: Smooth muscle active peptide					
**Genus: Euphlyctis**							
	*E. hexadactylus*	Crude skin extract: G+: 120–260 µg/mL G−: 120–520 µg/mL F: 32–64 µg/mL					

G+: gram positive bacteria; G−: gram negative bacteria; F: fungi; LD_50_: mean lethal dose against RBC; IC_50_: drug concentration causing 50% inhibition against cancer cell lines.

Amolopins, a novel family of two peptides (P1 and P2), were isolated from *A. loloesis* and have shown potent antimicrobial activity [15]. Both peptides were effective against the tested gram-positive bacteria over a range of 37–75 µg/mL, though they were found not to be effective against the tested range of gram-negative bacteria. Amolopins also showed 3% hemolysis at 200 µg/mL [15]. Additionally, Amolopin 1 and Amolopin 6 were isolated from *A. hainanensis* [10], and Jindongenin—a novel family characterized from *A. jindongensis—*were found to be effective against gram positive (Minimal inhibitory concentration [MIC]: 17–60 µM), gram-negative bacteria (MIC: 10–40 µM) and fungi (*Candida albicans*: MIC 60 µM). Cytotoxicity analysis of Jindongenin from *A. jindongensis* against K562 and HT29 revealed that IC_50_ values ranged between 40–46 µM [11].

Hainanenins 1–5 were isolated from *A. hainanensis* [10] and also showed potent antimicrobial function against gram-positive bacteria, gram negative bacteria and fungi in the ranges 4–40 µg/mL, 4–75 µg/mL, and 2–75 µg/mL respectively. Three Bradykinin related peptides (BRPs) named Amolopkinins were found in *A. loloesis* and *A. wuyiensis* [16,17]. Amylopkinin from *A. loloesis* exhibited dose dependent contractile activity in guinea pig ileum [16]. The function of BRPs in frog skin secretion is thought to relate to smooth muscle contraction activity—if a predator swallows the frog, these peptides induce smooth muscle contractions that will result in a vomiting sensation, thereby allowing the frog to escape predation. Two other Amylopkinins from *A. wuyiensis* inhibited Bradykinin induced contractile effects on isolated rat ileum smooth muscle preparations [17].

### 2.2. Clinotarsus

The Clinotarsus genus is endemic to the Western Ghats of India where new HDPs are being discovered. The peptidomic approach revealed the presence of five novel peptide amides homologous to the Brevinin 1 family from a single species, *C. curtipes*, that all show promising effectiveness against gram-positive and gram-negative bacteria [81]. The MICs against the tested pathogenic bacteria were reported to be between 6–25 μg/mL (Table 1 and Table 2). It was previously reported that bacterial membranes depolarize during pore formation and bacterial killing, but in the case of Brevinin 1 from *C. curtipes*, it was shown that depolarization and bacterial killing are independent events. We are slowly beginning to understand the methods by which these HDPs effectively fight disease.

### 2.3. Euphlyctis

Ramesh B. *et al.* [92] reported on the potential of HDPs discovered on the skin of the Indian green frog, *Euphlyctis hexadactylus*. They showed that the lyophilized crude skin secretion was inhibiting the growth of pathogenic gram-positive and gram-negative bacteria, as well as fungal pathogens—which included plant fungal pathogens. The MIC of the peptide against several human-relevant bacteria ranged from 128 to 512 μg/mL and for fungus it ranged between 32 to 64 μg/mL (Table 1 and Table 2). However it has not yet been reported what the composition of these skin secretions might be.

### 2.4. Fejervarya

Two Tigerinin peptides were identified from *Fejervarya cancrivora* [82], similar to the Tigerinins originally isolated from the Indian frog *Hoplobatrachus tigrinus*. The effects of a C-terminal amidation on both peptides include a decrease in MIC against pathogens compared to their non-amidated counterparts. MICs of non-amidated Tigerinins against gram-positive bacteria, gram-negative bacteria and fungi ranged from 20–80 µg/mL, 10–40 µg/mL and 80–180 µg/mL respectively (Table 1 and Table 2) and that of C terminally amidated peptides were 10–40 µg/mL, 5–40 µg/mL, and 15 µg/mL respectively. They also exhibited 10%–12% hemolysis against rabbit red blood cells (RBCs) at 100 µg/mL [82]. With this data we can better understand the influence of the peptide’s sequence on its function.

### 2.5. Glandirana

HDPs with significant antimicrobial activity have been reported from two species of genus Glandirana—*G. rugosus* [13,18] and *G. emeljanovi* [19] (Table 1). Novel peptides such as Rugocin A–C and Gaegurins 1–3 (6 peptides) were characterized from *G. rugosus*, which is now reclassified as the Brevinin 2 family [20]. Gaegurins 5–6 from *G. emeljanovi* have been reclassified as the Brevinin 1 family [20], and Gaegurin 4 as Esculentin 2 [19]. MICs of Brevinin-2 (Rugosin A and B) from *G. rugosus* were found to be 6–50 µg/mL for gram-positive bacteria and 12.5–100 µg/mL for gram-negative bacteria (Table 1 and Table 2) [18].

### 2.6. Hoplobatrachus

Tigerinins, first reported in *H. tigerinus*, [83] are a family of 4 peptides identified in *H. rugulosus* that are characterized by potent antimicrobial activity against gram-positive bacteria (MICs: 20–50 µg/mL) and gram-negative bacteria (MICs: 20–100 µg/mL) (Table 1 and Table 2). Tigerinins isolated from *H. rugulosus* have also been found to stimulate insulin release from rat BRIN-BD11 clonal β cell line [84,85]. Ojo *et al*. [84] also reported that C-terminal amidation was crucial for its activity.

### 2.7. Hyla

Anntoxin, a peptide that inhibits tetradotoxin-sensitive voltage-gated sodium channels, was identified in the skin secretions of *Hyla annectans*. This peptide is believed to have a great therapeutic potential as an anti-nociceptive and anti-inflammatory agent [86] (Table 1 and Table 2).

### 2.8. Hylarana 

Many of the peptides isolated from the different Hylarana species belong to the Brevinin 1 and 2, Esculentin 2, and Temporin families, and have showed activity against gram-positive and -negative bacteria, fungi and cancer cells (Table 1 and Table 2) [21,22,23,24,25,26,27,28,29,30]. Gaegurins (7 peptides) and Rugosins (9 peptides) from *H. nigrovittata* have been reclassified to the families Brevinin 1 and 2, respectively [27]. Brevinin 2 from *H. guentherii* was also able to stimulate insulin secretion from the rat BRIN-BDII clonal β cell line [25].

*H. nigrovittata* and *H. spinulosa* also produce a novel family of antimicrobial peptides [27,30] called the Nigroain family, which exhibited activity against gram-positive bacteria (MICs: 9–50 µg/mL), gram-negative bacteria (MICs: 25–110 µg/mL) and fungi (MICs: 2–4 µg/mL). Bradykinin-like peptides identified from this genus include Ranakinin N and Cholycytokinin from *H. nigrovittata*, as well as BRPs from *H. guentherii* [31,32,33]. Ranakinin N induced dose-dependent contractile effects on guinea pig ileum [32] and BRPs from *H. guentherii* revealed dose-dependent contraction of intestinal smooth muscle but did not have any effect on rat arterial smooth muscle [33].

Antimicrobial peptide Guentherin was first reported from *H. guentherii* and showed potent activity only against gram-positive bacteria (MIC: 33.5 µg/mL) [33]. Odorranain, another novel peptide of Odorrana genus, was identified from *H. spinulosa* [30]. Antimicrobial peptide Esculentin 1 was identified in three Hylarana species: *H. latouchii, H. spinulosa* and *H. luctuosa* [8,24,30]. Palustrin 2 was reported from *H. latouchii*, *H. signata* and *H. luctuosa* [24,29]. Palustrin 2, found on *H. latouchii,* was active only against gram-positive bacteria (MICs: 1–14 µM). Three peptide familes, Ranateurin, Ranacyclin, and Spinulosain were uniquely found in *H. spinulosa* [30]. These three peptides did not show any antimicrobial activity against the tested pathogens but Ranatuerin exhibited very low hemolysis of 10% at 200 µM against rabbit RBCs.

*H.temporalis* is the first among the genus to be reported from Western Ghats, India. Seven novel peptides are reported from *H.temporalis,* which include one Brevinin 1, two Brevinin 2 peptides, three Esculentin 2 peptides, and one Hylaranakinin peptide [34,35,36]. The MICs of Brevinin and Esculentin peptides ranges from 30 to 150 μg/mL.

### 2.9. Limnonectus

Limnonectins (2 peptides) from *L. fujianensis* are the only family of HDPs reported from the genus Limnonectus thus far [87] (Table 1 and Table 2). These peptides were reported to have no antimicrobial activity towards gram-positive bacteria and fungi, but MICs against gram-negative bacteria range between 35–70 µM. They exhibited no hemolysis up to a concentration of 160 µM.

### 2.10. Nanorana

Genus Nanorana has provided the antimicrobial peptides Japonicin 1 (2 peptides) and a novel family named Parkerin, both isolated from *N. parkeri* [88] (Table 1 and Table 2). Japonicin 1 and Parkerin peptides were reported to be effective only against gram-positive bacteria with MICs in the range 9–37 µg/mL and 37–40 µg/mL respectively. 1%–3% hemolysis was observed for both peptides at 80 µg/mL. Mast cell degranulation, which is thought to mediate antimicrobial activity of HDPs, was exhibited by both the peptides.

### 2.11. Odorrana

HDPs have been identified from ten species of Odorrana (Table 1). *O. grahamii* is one of the well-studied species in Asia. Common peptide families include Brevinin 1 and 2, Esculentin 1 and 2, and Nigrocin 2 [28,31,37,38,39,40,41,42,43,44,45,46,47], all of which have been shown to be effective against gram-positive and -negative bacteria, as well as fungi. A novel antimicrobial peptide family, Odorranain, was reported from four species of the genus [14,37,41,42,48,49]. MICs of Odorranain peptides against gram-positive bacteria, gram-negative bacteria and fungi were in the ranges 2–90 µg/mL, 3–50 µg/mL and 1–50 µg/mL, respectively. MIC of Odorranain towards *Helicobacter pylori* was reported to be 20 µg/mL (Table 1 and Table 2).

Many novel peptide families were reported from *O. grahamii* such as Tachykinin (2 peptides), Odorranalectin (the smallest lectin reported so far), 5 Bombesin-like peptide, and 2 Grahamins [37,48,49,50,51,52]. Odorranalectin coupled with a nanoparticle complex improved the therapeutic effects of UCN-loaded nanoparticle in treating Parkinson’s disease. Results also suggested the use of Odorranalactin as a carrier in nasal delivery of macromolecular drugs to the brain [53]. Antimicrobial activity of Grahamins was reported against gram-positive bacteria (MIC: 2.5 µg/mL), gram-negative bacteria (MICs: 1–8 µg/mL) and fungi (MIC: 7.5 µg/mL). Trypsin inhibitor peptide was isolated from *O.grahamii*, *O. versablis*, and *O. hejiangensis.* These inhibitors are thought to protect the host from a range of proteases produced by invading pathogens [47,54,55].

Studies of the skin of *O. tiannanensis* also revealed a set of novel HDP families—Margaretaein (2 peptides), Pleurain, and Tiannanensin [42]. Tiannanensin, an antimicrobial peptide, showed activity against gram-positive bacteria and fungi with MICs 75 and >100 µM, respectively. In addition to these there are other novel HDP families including Iksikawain, Lividin (first report), and Ranateurin, found in *O. ishikawae*, *O. livida*, *and O. versablis*, respectively [41,43,56]. Bradykinin related peptides were also isolated from *O. grahamii* (3 peptides) and *O. schmakeri* (7 peptides) [57,58]. Three of the five Bombesin-like peptides from *O. grahamii* induced contraction of the stomach muscle tissue. The other two BLPs, which have a C terminal octapeptide, antagonized stomach muscle contraction. Inhibition of contraction is thought to be due to the presence of the octapeptide [37].

### 2.12. Pelophylax

Five species of the genus Pelophylax (*P. plancyi*, *P. porosus*, *P. chosensicus*, *P. nigromaculata*, and *P. fukienensis*) are well-known for their HDPs (Table 1 and Table 2). Pelophylaxins 1–4 are a novel peptide group with no orthologs, and are found in *P. fukienensis* [56]. Two other novel peptides, Nigrocin 1 and 2, were isolated from *P. nigromaculata* [13,59] and later reclassified to the Brevinin 2 family [20]. Brevinin 2 (Nigrocin 1) was reported to have antimicrobial activity towards gram-positive bacteria (MIC: 2.5 µg/mL), gram-negative bacteria (MIC: 10–100 µg/mL), and fungi (MIC: 100 µg/mL). Nigrocin 2 was also reported to be effective against gram-positive and gram-negative bacteria as well as fungi in the ranges 2.5 µg/mL, 10–200 µg/mL, and 150 µg/mL, respectively. Both of the peptides from *P. nigroemaculata* showed low hemolysis of 0.9%–1% at 100 µg/mL. A Bradykinin antagonist named Ranakenestatin was recently reported from *P. fukienensis*—though the exact function of this peptide in frogs remains controversial, it is expected to have great potential in therapeutic applications [60].

### 2.13. Rana

Rana is one of the most diverse and well-studied of the amphibian genera in Asia with respect to their host defense peptides. HDPs have been isolated from 16 different species (Table 1 and Table 2). Common antimicrobial peptide families that were identified from this genus include Brevinin 1 and 2, Ranateurin, and Temporin 1 [61,62,63,64,65,66,67,68,69,70,71,72,73,74,75]. Temporin 1 from *R. chensinensis* exhibited cytotoxic effects against 12 tested carcinoma cell lines. They also showed low hemolytic effect against human RBCs and no considerable cytotoxic activity against normal human umbilical vein smooth muscle cells (HUVSMCs).

Japonicin 1 and 2, first isolated from *R. japonica*, were isolated from three other species of the genus [62,64,65,74]. Japonicins were found to be effective against gram-positive and gram-negative bacteria in the range of 25–100 µg/mL and 12–100 µg/mL respectively. They were also reported to be strongly hemolytic against mammalian RBCs.

Amurins reported from *R. amurensis* and Dybowskins from *R. Dybowsskii* (1–6: 6 peptides) [62] have been reclassified to the Brevinin 1 family [20]. Palustrin 1, first identified from *R. palustris*, was also reported from *R. ornativentris* [67]. Mellitin related peptides (MRPs) were isolated from *R. tagoi* and *R. sakuraii* [70,76]. MRPs are active against gram-positive bacteria, gram-negative bacteria, and fungi. Bradykinin related peptides were reported from *R. okienensis*, *R. tagoi*, *R. chensinensis* and *R. sakuraii* [69,71,72,77]. Shuchin (5 peptides) and Pleuran, two novel peptide families, were identified from *R. shuchinae* and *R. pleuridan*, respectively, both of which are potent against gram-positive and -negative bacteria, as well as fungi [78,79,80].

### 2.14. Rhacophorus

HDPs from two species—*Rhacophorus duboisi* (previously *Polypedates pingbianensis*) and *Rhacophorus schegelii*—have recently been isolated (Table 1 and Table 2). Polypedarelaxin from *Rhacophorus duboisi* effectively relaxes smooth muscle contractions [89]. This peptide exhibited concentration-dependent relaxation effects on isolated rat ileum but did not show antimicrobial and serine protease activity. Cationic antimicrobial peptides do not appear in the dermal immune system of *Rhacophorus schegelii* [90]. However, Histone 2B was reported from *Rhacophorus schegelii*, [90] which is evidence for the role of histones as antimicrobials in addition to their ability to remodel chromatin. This was the first report of histones from frog skin secretion.

### 2.15. Sanguirana

HDPs isolated from *Sanguirana varians* (Table 1 and Table 2) of the genus Sanguirana revealed homology to the neuroendocrine peptide Bombesin [91]. These BLPs revealed dose dependent contractile effects of stomach tissue.

## 3. Conclusions

With the increasing emergence of multi-drug resistant pathogens, as well as the constant struggle against non-infectious illnesses such as cancer, there is a critical need to develop new therapies where old treatments have failed. Amphibian-derived antimicrobial peptides offer us a potential solution. We are now just beginning to understand the diversity and functions of these peptides, but this review outlines the ways in which we already see the potential clinical uses of these HBPs. The potent antimicrobial peptides in Asian frogs belong to the Brevinin and Esculentin. They are very effective against gram-positive and negative-bacteria as well as fungi. The only problem with these peptides is their high cytotoxicity, which would be eliminated by effective modification. The Temporin family of peptides are potent anticancer agents which are highly toxic against cancer cells, especially breast cancer cell lines and demonstrate very low toxicity towards normal cells (e.g., temporin from *R. chensinensis*). Hopefully further study of these animals and their skin peptides will reveal novel therapies that may potentially fill the gap left by antibiotic resistance and the failure of many of our current cancer treatments. There is therefore a need to turn our attention to this region and its biodiversity as a key medical resource.
